# Piezoresistive Conductive Microfluidic Membranes for Low-Cost On-Chip Pressure and Flow Sensing

**DOI:** 10.3390/s22041489

**Published:** 2022-02-15

**Authors:** Md. Nazibul Islam, Steven M. Doria, Xiaotong Fu, Zachary R. Gagnon

**Affiliations:** 1 Artie McFerrin Department of Chemical Engineering, Texas A & M University, College Station, TX 77843, USA; nazibul@tamu.edu (M.N.I.); sdoria1@tamu.edu (S.M.D.); 2 Department of Chemical and Biomolecular Engineering, Johns Hopkins University, Baltimore, MD 21218, USA; xiaotong.fu0826@gmail.com

**Keywords:** microfluidics, impedance spectroscopy, pressure sensor

## Abstract

Over the last two decades, the field of microfluidics has received significant attention from both academia and industry. Each year, researchers report thousands of new prototype devices for use in a broad range of environmental, pharmaceutical, and biomedical engineering applications. While lab-on-a-chip fabrication costs have continued to decrease, the hardware required for monitoring fluid flows within the microfluidic devices themselves remains expensive and often cost-prohibitive for researchers interested in starting a microfluidics project. As microfluidic devices become capable of handling complex fluidic systems, low-cost, precise, and real-time pressure and flow rate measurement capabilities have become increasingly important. While many labs use commercial platforms and sensors, these solutions can often cost thousands of dollars and can be too bulky for on-chip use. Here we present a new inexpensive and easy-to-use piezoresistive pressure and flow sensor that can be easily integrated into existing on-chip microfluidic channels. The sensor consists of PDMS–carbon black conductive membranes and uses an impedance analyzer to measure impedance changes due to fluid pressure. The sensor costs several orders of magnitude less than existing commercial platforms and can monitor local fluid pressures and calculate flow rates based on the pressure gradient.

## 1. Introduction

To carry out complex on-chip fluidic applications, including drug screening, medical diagnostics, chemical analysis, and environmental monitoring, microfluidic platforms must be capable of controlling on-chip flows during pumping, routing, and mixing operations [1,2,3,4]. Flow sensors that detect the flow rate of fluid locally within microfluidic channels are therefore essential components for lab-on-a-chip (LOC) systems. The most established commercially available sensors for measuring microfluidic flows are large off-chip flow sensors which measure external fluid flow rates at the inlets and outlets of the fluidic device. External sensors, however, are often bulky and cannot be integrated within a microfluidic channel to locally detect fluid flows and local pressure gradients. Furthermore, most commercial flow sensors have large dead volumes and are expensive, costing upwards of USD 1000 per sensor. With the increasing complexity and demand for portable LOC systems, a small, scalable, low-cost microscale flow sensor is becoming an important component for LOC systems that require precise flow control and fluidic routing.

Currently, there is a lack of small low-cost flow sensors that can be scalably integrated into a network of microfluidic channels. Such sensors could enable improved methods for controlling and metering fluid flows and help increase the portability of micro total analysis systems. Efforts have been made to develop optical sensors that detect and measure micro-scale fluid flows on-chip. In 2004, Kohl et al. linked the reflection angle of incident light. Light was reflected off an optical lever atop a thin PDMS to sense the internal pressure within the microfluidic channel [5]. In 2011, Song and Psaltis measured optical interference induced by pressure-driven deformations in a thin PDMS layer that separated an open channel from a microfluidic flow channel [6]. In 2017, Christian et al. leveraged an oxygen-sensitive fluorescent indicator to measure the compression of air within a cavity adjacent to a microfluidic flow channel [7]. While such optical sensors tend to be effective over a wide range of pressures, they require bulky microscopy setups, which are not compatible with point-of-care applications.

Several electrical methods exploit the fact that the internal pressure within an LOC device can deform a flexible membrane, and can be utilized to detect changes in electrical properties of the deforming walls surrounding the flow channel. In 2009, Li et al. fabricated periodic Ni-PDMS composite posts inside of a microfluidic flow channel and linked the resistivity between posts to local pressures within the device [8]. In 2017 Wang et al. implemented a piezoelectric sensor to measure pressure changes in an air channel separated from a microfluidic flow channel by a thin membrane [9]. Other approaches include nested ionic circuits atop a microfluidic flow channel [10,11]. While precise, such devices require a tedious multi-layer fabrication. In 2020, Peng et al. measured a deformation-induced resistivity change in a membrane separating a main flow channel from two ionic electrodes [12]. Although effective, such devices induce very small changes in resistivity over large changes in pressure, sacrificing detection precision for a larger measurable pressure range.

In this article, we report a new portable, cost-efficient, scalable, and sensitive microfluidic sensor capable of measuring the local on-chip pressure and flow rate in a microchannel. The sensor is simple to fabricate; we combine conductive carbon black powder with a polydimethylsiloxane (PDMS) elastomer and integrate this nanocomposite mixture directly into the sidewalls of microfluidic channels using a novel soft lithography technique. When fluid flow is driven within the channel using external pressure, the PDMS membranes exhibit a piezoresistive behavior. By measuring the electrochemical impedance spectroscopy (EIS) of these membranes while under stress, we show that the flow rate and pressure drop within the microchannel can be precisely captured in real-time. In the first part of this paper, we discuss the working principle of our flow sensors. We then describe the fabrication process for our sensors. Next, we present the influence of hydrostatic microchannel pressures on the electrical conductivity of our carbon black membranes using EIS. We then develop a COMSOL model for membrane deformation and an equivalent circuit model for the sensing unit. Lastly, we integrate two sensors into a single microfluidic channel and perform flow rate measurements. We compare our sensor measurements to flow data collected using state-of-the-art commercially available flow meters that cost more than USD 1000 per device and show similar performance with our fabricated sensors that cost less than USD 2. We show that that our presented sensor design allows for microfabricated flow meters to be embedded into any PDMS-based microfluidic device to monitor pressure changes in real time without interrupting the local fluid flow field. Because of their small size, it is possible to integrate multiple units within a single microfluidic channel to sensitively measure local pressure gradients and fluid flow rates at a cost several orders of magnitude less than current external flow sensors.

## 2. Experimental

### 2.1. Working Principle of Piezoresistive Sensor

The microfluidic pressure sensor consists of a pair of deformable polydimethylsiloxane–carbon black (PDMS/CB) composite membranes integrated into the wide walls of a microchannel and an external electrochemical impedance spectroscopy (EIS) analyzer. The PDMS/CB membranes are fabricated directly into the sidewalls of the microfluidic channel. Fluid flow is driven into this channel using an axial pressure gradient generated by an external pressure source. When the flow channel is pressurized, local pressure within the channel exerts a normal stress on the channel sidewalls, forcing the PDMS/CB membranes to deform outward perpendicular to the direction of flow. This outward deformation then influences the electrical conductivity and capacitance of the conductive sidewall membranes. This change in membrane impedance can then be transduced to an external EIS analyzer using microfabricated on-chip gallium metal electrodes. The variation in membrane resistance is proportional to the membrane deformation and the local pressure inside the flow channel. By correlating the impedance measurements to the applied pressure, we can measure the pressure changes inside the main flow channel in real-time. To monitor the pressure gradient and corresponding microchannel flow rate, two pressure-sensing units were embedded in both ends of a microfluidic flow channel and the pressure gradient was calculated using EIS. The pressure gradient was then converted into a flow rate using the hydrodynamic resistance between the two sensors.

### 2.2. Sensor Microfabrication

**Figure 1:**
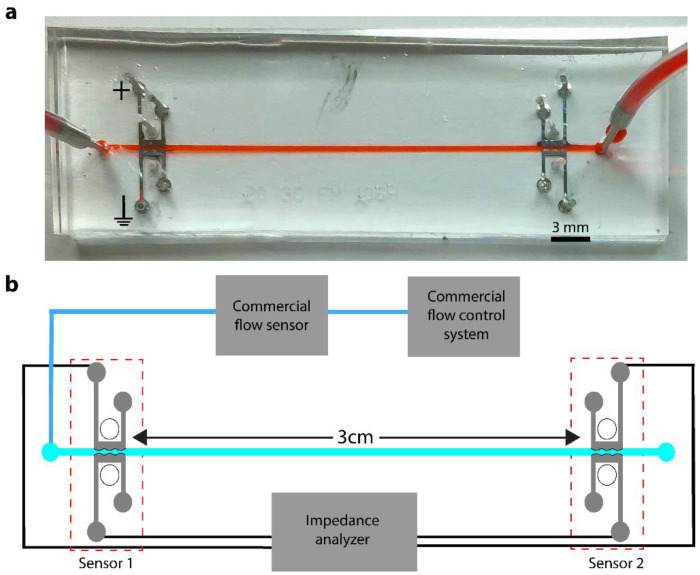
(**a**) The piezoresistive microfluidic sensor for real time pressure and flow rate determination. (**b**) The schematic diagram and working mechanism of the pressure and flow sensor.

The microfluidic device consists of a main flow channel with two separate integrated pressure sensors to detect the pressure gradient. Each sensing unit is fabricated into the channel sidewalls and contains two metal electrodes separated from the flow channel by a PDMS/CB membrane (Figure 1a). To locally pattern each sidewall membrane, a previously reported multistage soft lithographic process was used [13]. Briefly, the soft lithographic microchannel mold was fabricated using a negative photoresist, SU-8 3050 (Microchem Corp., Newton, MA, USA). The main flow channel is 400 μm in width, 80 µm in height, and 3 cm in length. Each sensing unit consists of two gallium electrode channels separated from the main flow channel by a 1300 μm long and 20 μm thick gap filled with PDMS/CB nanocomposite elastomer. The carbon black nanocomposite (Sigma-Aldrich, 633100, St. Louis, MO, USA) was combined with a 1:4 weight ratio of PDMS elastomer and mixed in a centrifugal mixer for 30 s (Thinky, ARE-310, Laguna Hills, CA, USA). The resulting gel-like elastomer mixture was then patterned into each gap between the channels. After removing the excess gel using a razor blade, a 1:10 mixture of PDMS elastomer and curing agent was poured atop the mold and allowed to cure for an hour at 80 °C, during which time the entire elastomeric system cured. The cured PDMS slab was gently peeled off the mold and the channel inlet and outlet fluid ports were hole punched using a 0.75 mm biopsy punch (Ted Pella, Inc., Redding, CA, USA). In addition, to enhance electrode deformation, 0.75 mm holes were punched at 85 µm from the electrodes (Figure 1b). The resulting PDMS device was then bonded to a glass coverslip by exposure to oxygen plasma and immediately aligned and sealed under an inverted brightfield microscope. Finally, the chip was baked for 24 h at 80 °C to enhance the bond strength. To fabricate each metal electrode, solid gallium metal (Sigma-Aldrich, 263265) and the PDMS chip were heated to 40 °C on a hot plate. With a melting temperature of 29.7 °C, the newly melted liquid gallium was loaded into a 1 mL plastic syringe and immediately injected into the electrode channels. The electrical connection was made using 0.75 mm diameter copper wire leads inserted into each electrode injection hole.

### 2.3. Device Experimental Setup and Operation

Initial operations consisted of priming the main flow device with a common electrolyte and subjecting the microfluidic channel to known pressures. 1X PBS was used as the working solution for all the sensor characterizations and tests. Briefly, the PBS solution was driven by a constant pressure flow system (Elveflow, OB1, Paris, France) and directed into the main flow channel (Figure 1b). A commercial flow sensor (Elveflow, MFS, Paris, France) was attached to the inlet for real time flow rate measurement during sensor characterization and calibration. An electrical connection was made between the gallium electrodes and the impedance spectrometer (Agilent, HP 4192A, Santa Clara, CA, USA) through copper wire leads, which were inserted into the gallium electrode ports. Two separate LabVIEW scripts were used to perform impedance measurements. The first script measured the magnitude of impedance (|Z|) and angle (θ) while sweeping the applied frequency. The second script measured both |Z| and θ, while holding the applied frequency constant. Equivalent circuit modelling was performed using ZView software.

## 3. Results and Discussion

### 3.1. Sensor Impedance Response

**Figure 2:**
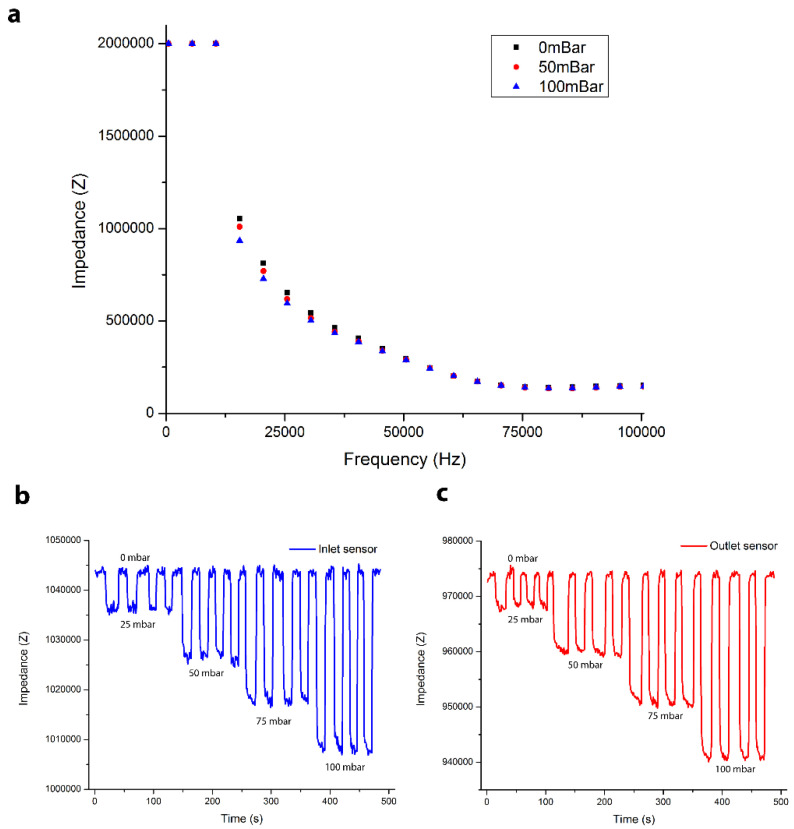
(**a**) The impedance response at different frequencies for different inlet pressures. (**b**) The inlet sensor impedance response at 15.5 kHz for different inlet pressures. (**c**) The outlet sensor impedance response at 15.5 kHz for different inlet pressures.

The external impedance analyzer used in this study had a working frequency bandwidth from 0 Hz to 13 MHz. To achieve improved device sensitivity, the operating (excitation) frequency was optimized by running frequency sweeps over different applied pressures in order to determine the optimal operating frequency for maximized pressure sensitivity. A LabVIEW script was used to sweep a frequency range, from 500 Hz to 200 kHz, and plot the measured impedance response to frequency change. The experiment was repeated for three different applied pressures, 10 mbar, 50 mbar, and 100 mbar, as shown in Figure 2a. Under low frequency, the AC field is not capable of overcoming the side wall membrane capacitance and the corresponding impedance was larger than the upper reading limit of the impedance analyzer (2 MΩ), as shown in Figure 2a. As the excitation frequency increased, the frequency-dependent capacitance of the membrane is capable of being surpassed and the current penetrates the PDMS/CB membrane. The corresponding impedance then drops with increasing frequency and eventually reaches an approximate constant value. In terms of optimizing the sensor performance, the greatest impedance difference was determined to exist at the applied frequency of 15.5 kHz, which was then used as the optimum operating frequency for all subsequent pressure sensing experiments. Note that at high frequency ranges, the impedance becomes very small, thus the difference in impedance between different applied pressures is barely noticeable, Figure 2a.

Next, the pressure sensor was characterized and calibrated to correlate the impedance measurements with local pressure changes by plotting step curves at different inlet pressures for a constant frequency, Figure 2b,c. As mentioned earlier, a frequency of 15.5 kHz was applied, and the external pressure source was varied from 0 mbar to 100 mbar. The outlet was blocked to achieve uniform pressure across the entire channel. The resulting impedance variation of the inlet and outlet sensors was recorded in real time, as shown in Figure 2b,c. There is a 7% variance in data between the two sets of sensors. Manual injection of liquid gallium and PDMS/CB composite might introduce a slight variation in the sensor surface area, resulting in sensor data variance. At each pressure, quadruplicated data sets were recorded, and each set contains 10 to 15 data points. To achieve better accuracy and avoid external interferences, OriginPro data analysis software was used to create a baseline according to the impedance reading at 0 mbar. Thus, all the data points at 0 mbar were normalized to the same level after subtracting the baseline. This step guarantees that all the impedance readings have the same reference point; therefore, impedance changes are only caused by pressure variation inside the flow channel.

### 3.2. Finite Element Modelling

**Figure 3:**
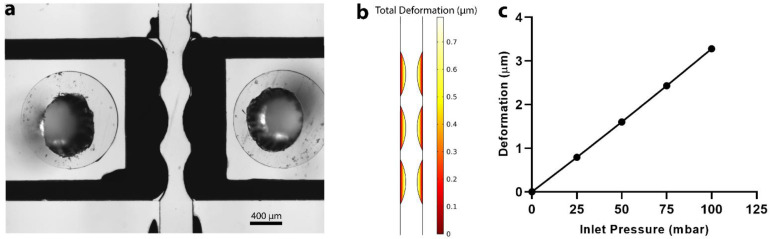
(**a**) The pressure sensing unit. (**b**) The COMSOL representation of the pressure sensing unit with membrane deformation. (**c**) The membrane surface displacement at different inlet pressures.

A finite element model was developed using the numerical solution of the equations of motion within the microfluidic channel. As fluid flows across the sensor membrane sidewalls, and a membrane stress is induced on the sensor, deforming the PDMS/CB membrane. This deformation can be determined using the Arbitrary Lagrangian–Eulerian (ALE) Finite Element Method. Fluid flow can be modelled using the Navier–Stokes equation and solid structure interactions can be modelled using a two-dimensional equation of motion. The solid and fluid structures can be coupled by using kinematic and dynamic continuity boundary conditions at the interface. The membrane deformation model was solved numerically using a finite element package (COMSOL Multiphysics, 5.4) with an element mesh consisting of 23,878 triangular and 2168 quadrilateral mesh elements per microfluidic channel. For this study, the geometry of the sensing unit was comprised of a thin PDMS/CB membrane, a gallium electrode, and a circular hole in the PDMS structure adjacent to the electrode, as shown in Figure 3a. This two-dimensional (2D) geometry was created in COMSOL, Figure 3b. A Young’s modulus of 145 kPa and a Poisson’s ratio of 0.4 were used as the effective membrane stiffness. These values are within the range of published data [14,15]. The effective stiffness takes into account the PDMS/CB membrane, gallium electrode, and PDMS structure deformation due to the pressure applied by fluid flow and is used to represent the actual sensing unit. All the other material parameters were unchanged and used as defined by COMSOL’s materials database. The inlet pressure of the fluid flow of the model was increased, and the resultant deformation (maximum displacement) of the sensing unit was calculated. As shown in Figure 3c, deformation is predicted to increase linearly with an increase in inlet pressure over the experimental pressure range used in this work. This deformation will then change the electrical resistivity of the conductive membrane sensing unit, which can be transduced by the impedance analyzer, and a calibration curve of the impedance response to a change in inlet pressure can be developed.

### 3.3. EIS Equivalent Circuit Model

**Figure 4:**
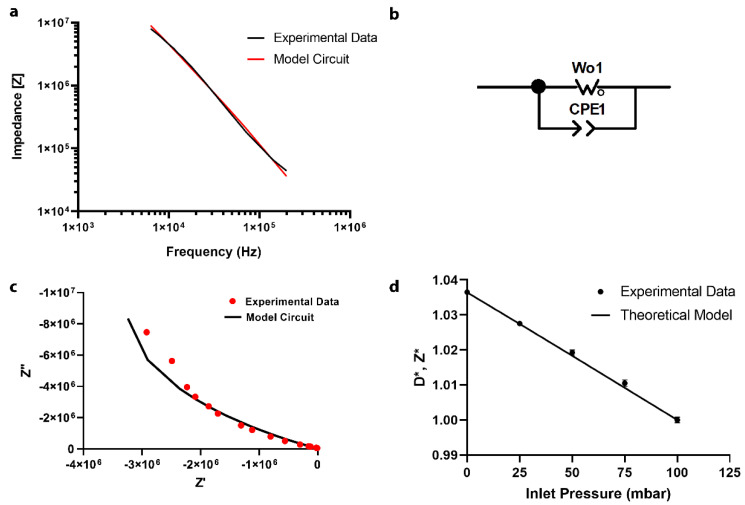
(**a**) The Bode plot for the experimental data, the equivalent circuit model, and the Bode plot for the equivalent circuit. (**b**) the equivalent circuit model based on the Bode plot. (**c**) The Nyquist plot for the equivalent circuit model (**d**) The change in dimensionless impedance (experimental data) and dimensionless deformation (theoretical model) for different inlet pressures (one standard deviation error bar for *n* = 5 measurements).

An equivalent circuit model was developed using electrical impedance spectroscopy (EIS) to investigate the change in sidewall membrane impedance upon deformation. Several different equivalent circuit models were tested by non-linear least-squares fitting using ZView software to determine the best-fit model for experimental impedance data (Figure 4a). As shown in Figure 4b, an equivalent circuit consisting of a finite Warburg element (*W_o_*) and a constant phase element (CPE) in parallel provide a strong fit for the applied frequency range (χ^2^: 0.017). The *W_o_* element represents the network of resistive-capacitive (RC) ladder networks at the electrode/electrolyte interface, driven by charge transport across the diffuse double layer at the membrane-fluid interface [16]. This Warburg impedance element has been previously reported in circuit models containing PDMS–zeolite and poly (3,4 ethylenedioxythiophene) (PEDOT) film electrodes [17,18] and can be represented by the following equation:

$$Z_{W_{o}}=Z_{o}{\left(j\omega \tau \right)}^{-p}coth{\left(j\omega \tau \right)}^{p}$$

where *Z_o_* is the Warburg constant, *τ* is diffusion time constant, and *p* is a value between 0 and 1 [19]. The constant phase element (CPE) appears as a double-layer capacitance on a surface and can be represented by the following equation:

$$Z_{\mathrm{CPE}}=\frac{1}{Q_{o}{\left(j\omega \right)}^{n}}$$

where, *Q_o_* is the CPE capacitance and *n* is a value between 0 and 1 [19]. When *n* equals 1, CPE behaves as an ideal capacitor. The appearance of CPE in our system represents double-layer polarization on the surface of the electrodes on each side of the microchannel walls. To access the quality of the equivalent circuit model, a Nyquist plot was developed to compare the experimental values of the real and imaginary portion of the impedance data with that of the theoretical model (Figure 4c). The Nyquist plot shows that the experimental data are in good accordance with the equivalent circuit model. When an AC electric field is applied across the channel, the ions in the solution electromigrate and accumulate on the surface of the conductive PDMS/CB membrane, creating an induced electric double layer (EDL). The diffusion element represents ionic flux into and out of the EDL.

### 3.4. Impedance Response to Membrane Deformation

As shown in Figure 3c, an increase in inlet pressure results in a linear increase in the deformation of the sensing unit. This deformation length scale is two orders of magnitude greater than the typical electrical double-layer length scale [20]. Therefore, a change in impedance at a given excitation frequency is largely not influenced by EIS itself, but rather by the physical deformation of the resistive element. The resistivity of PDMS/CB has been reported to be several orders of magnitude higher than both gallium and 1X PBS [13,21,22]. Therefore, any change in sensor resistance will be due to the deformation-induced change in PDMS/CB membrane resistance. As the membrane deforms with increasing inlet pressure, the length of the resistor decreases, and the cross-sectional area increases, leading to a decrease in membrane resistance. This is represented by a drop in overall impedance, which can be represented by the following scaling relation:

$$\left[Z\right]\propto \rho_{\mathrm{PPDMS/CB}}\propto \frac{1}{Deformation},$$

where [*Z*] is the system impedance and *ρ*_PDSM/CB_ is membrane resistivity. Therefore, a linear increase in membrane deformation due to increasing inlet pressure will result in a linear decrease in impedance, as shown in Figure 4d. In Figure 4d, values in the *y*-axis were non-denationalized to match the impedance data (experimental) with the deformation data (model), where Z* and D* are represented by the following equations:

$$Z^{*}=\frac{Z}{Z_{100\;\mathrm{mbar}}}\;\mathrm{and}\;D^{*}=\frac{1/L}{1/L_{100\;\mathrm{mbar}}}$$

Here, *Z* is impedance and *L* is the distance between the two membranes.

### 3.5. Flow Rate Measurement

**Figure 5:**
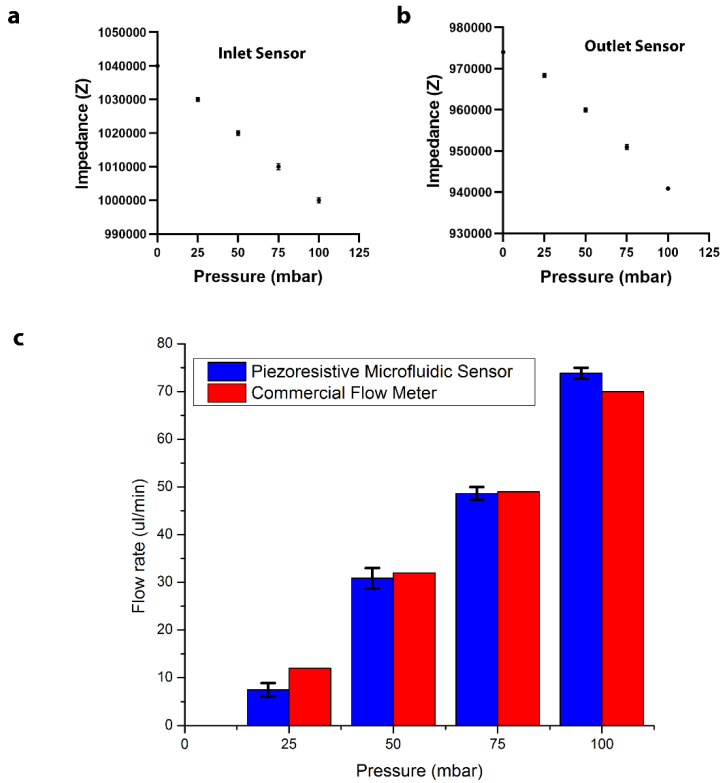
(**a**) The calibration curve for the inlet sensor. (**b**) The calibration curve for the outlet sensor. (**c**) A comparison of the measured flow rate between the piezoresistive microfluidic sensor and a commercially available sensor (one standard deviation error bar for *n* = 5 measurements).

One advantage of this newly designed sensor is that it can be integrated directly to the microfluidic channel sidewalls without impeding or blocking fluid flow. This allows the sensor to be applied to any existing microfluidic network to monitor the local pressure gradient or flow rate. Compared to current commercial flow meters, this new sensor has the capability for on-chip integration, allowing for a much smaller sensor size, as shown in Figure 1. To determine the flow rate, two sensors were integrated at the inlet and outlet regions of a straight microfluidic channel. Each sensor can monitor the local pressure changes individually. The average impedance for different inlet pressures was calculated and plotted for the inlet and outlet sensors to develop calibration curves (Figure 5a,b). For a given inlet pressure, the pressure gradient between the two sensing points was determined using the calibration curves. The corresponding flow rate inside the microchannel was calculated using the microchannel hydrodynamic resistance and compared to the flow rates measured by an external commercially available flow sensor. As shown in Figure 5c, the measured flow rates vary from 10 μL/min to 80 μL/min, similar to the commercial flow meter with 1–2 μL/min deviation.

## 4. Conclusions

We have developed an on-chip cost-efficient piezoresistive microfluidic sensor that is capable of obtaining both pressure and flow rate measurements. Compared to the current flow meters that cost more than USD 1000 per device, this newly developed sensor has a fabrication cost that is less than USD 2. The sensor contains two integrated conductive PDMS films on the microfluidic side wall. An impedance analyzer is connected to the elastic PDMS film through gallium metal electrodes. The film deformation caused by internal pressure changes can be translated into electrical resistance variations and then read by the impedance analyzer. This design allows the sensor to be integrated into any microfluidic device, monitoring pressure changes or measuring flow rates in real-time without interrupting the fluidic flow. In contrast to the previous piezoresistive microfluidic sensors, this newly designed sensor exhibits a simple fabrication process, low cost, on-chip integration, and the capability to monitor both pressure and flow rate in real-time. We characterized the sensor to determine the linear relation between pressure and impedance changes. Impedance value decreased linearly by increasing the applied pressure from 0 to 100 mbar. We developed a COMSOL model for membrane deformation and an equivalent circuit model for the sensing unit, showing that increasing the inlet pressure increases membrane deformation, leading to a decrease in sensor resistance and the resultant impedance. Lastly, we developed the actual flow meter with two integrated sensors and applied it to a microfluidic chip. The flow rates at a variety of external pressures were measured and compared to the flow rates measured using a commercial flow meter. The flowrates measured using the PDMS/CB piezoresistive sensor were comparable to those obtained by the commercial flow meters. One possible limitation of the flow sensor is sensor instability at a temperature higher than the melting point of gallium (30 °C).

Considering cost efficiency and the capability for on-chip integration, this newly developed piezoresistive microfluidic sensor can significantly improve real-time fluidic monitoring for use in increasingly complicated microfluidic systems.

## References

[1] Seo M., Paquet C., Nie Z., Xu S., Kumacheva E. (2007). Microfluidic consecutive flow-focusing droplet generators. Soft Matter.

[2] Gagnon Z., Mazur J., Chang H.C. (2010). Integrated AC electrokinetic cell separation in a closed-loop device. Lab Chip.

[3] Gagnon Z.R. (2011). Cellular dielectrophoresis: Applications to the characterization, manipulation, separation and patterning of cells. Electrophoresis.

[4] Fu X., Mavrogiannis N., Ibo M., Crivellari F., Gagnon Z.R. (2017). Microfluidic free-flow zone electrophoresis and isotachophoresis using carbon black nano-composite PDMS sidewall membranes. Electrophoresis.

[5] Kohl M.J., Abdel-Khalik S.I., Jeter S.M., Sadowski D.L. (2005). A microfluidic experimental platform with internal pressure measurements. Sens. Actuators A Phys..

[6] Song W., Psaltis D. (2011). Optofluidic membrane interferometer: An imaging method for measuring microfluidic pressure and flow rate simultaneously on a chip. Biomicrofluidics.

[7] Hoera C., Kiontke A., Pahl M., Belder D. (2018). A chip-integrated optical microfluidic pressure sensor. Sens. Actuators B Chem..

[8] Li H., Luo C.X., Ji H., Ouyang Q., Chen Y. (2010). Micro-pressure sensor made of conductive PDMS for microfluidic applications. Microelectron. Eng..

[9] Wang Z., Tan L., Pan X., Liu G., He Y., Jin W., Li M., Hu Y., Gu H. (2017). Self-Powered Viscosity and Pressure Sensing in Microfluidic Systems Based on the Piezoelectric Energy Harvesting of Flowing Droplets. ACS Appl. Mater. Interfaces.

[10] Wu C.Y., Liao W.H., Tung Y.C. (2011). Integrated ionic liquid-based electrofluidic circuits for pressure sensing within polydimethylsiloxane microfluidic systems. Lab Chip.

[11] Jung T., Yang S. (2015). Highly stable liquid metal-based pressure sensor integrated with a microfluidic channel. Sensors.

[12] Peng K., Yao J., Cho S., Cho Y., Kim H.S., Park J. (2020). Liquid metal embedded real time microfluidic flow pressure monitoring sensor. Sens. Actuators A Phys..

[13] Fu X., Gagnon Z. (2015). Contactless microfluidic pumping using microchannel-integrated carbon black composite membranes. Biomicrofluidics.

[14] Dogru S., Aksoy B., Bayraktar H., Alaca B.E. (2018). Poisson’s ratio of PDMS thin films. Polym. Test..

[15] MichelleáGrandin H. (2007). Micro-well arrays for 3D shape control and high resolution analysis of single cells. Lab Chip.

[16] Wang J.C. (1987). Realizations of Generalized Warburg Impedance with RC Ladder Networks and Transmission Lines. J. Electrochem. Soc..

[17] Bobacka J., Lewenstam A., Ivaska A. (2000). Electrochemical impedance spectroscopy of oxidized poly(3,4-ethylenedioxythiophene) film electrodes in aqueous solutions. J. Electroanal. Chem..

[18] Matysik S., Matysik F.M., Schulze K.D., Einicke W.D. (2002). Impedance spectroscopic investigations of zeolite–polydimethylsiloxane electrodes. Electrochim. Acta.

[19] Scribner Associates I.Z. View Impedance/Gain Phase Graphing and Analysis Software Operating Manual, Version 3.5. https://www.scribner.com/software/68-general-electrochemistr376-zview-for-windows/.

[20] Jurado L.A., Espinosa-Marzal R.M. (2017). Insight into the Electrical Double Layer of an Ionic Liquid on Graphene. Sci. Rep..

[21] Chaparro C.V., Herrera L.V., Meléndez A.M., Miranda D.A. (2016). Considerations on electrical impedance measurements of electrolyte solutions in a four-electrode cell. J. Phys. Conf. Ser..

[22] Ginter G., Gasser J.G., Kleim R. (2006). The electrical resistivity of liquid bismuth, gallium and bismuth-gallium alloys. Philos. Mag. B.

